# Ground beetles in Mediterranean olive agroecosystems: Their significance and functional role as bioindicators (Coleoptera, Carabidae)

**DOI:** 10.1371/journal.pone.0194551

**Published:** 2018-03-20

**Authors:** Roberto Pizzolotto, Antonio Mazzei, Teresa Bonacci, Stefano Scalercio, Nino Iannotta, Pietro Brandmayr

**Affiliations:** 1 Dipartimento B.E.S.T., Università della Calabria, Rende, Cosenza, Italy; 2 Centro di Ricerca Foreste e Legno, Rende, Cosenza, Italy; 3 ex C.R.E.A. Centro di Ricerca per l’Olivicoltura e l’Industria Olearia, Rende, Cosenza, Italy; Universidade Federal de Vicosa, BRAZIL

## Abstract

The impact of agricultural practices and soil management on the communities of arthropods living in the agricultural landscape is acknowledged as a critical issue by the literature, and it needs to be better investigated to improve the ecological sustainability of agriculture. In the present study, we aimed to study how soil management affect carabid species distribution in one of the most typical agroecosystem of the Mediterranean region, i.e. the olive grove. In South Italy olive plantations feature different types of soil management, from tillage to half- or full-cover cropping. Species distribution has been examined for a total of 10,189 individuals and 62 species collected from 17 sites. Notably from our analysis we have observed that three factors (climax vegetation, soil features and soil management) explained half of the data variability. The composition of species groupings mirrors both bioclimatic conditions (climax vegetation) and soil features, especially watering, while soil management affects the species distribution, with different intensity from site to site. Eleven species have been recognized as the most abundant in the different facets of the studied olive groves and consequently designated as characteristics of the olive agroecosystem. The species traits of the sampled species have been weighted for a compelling evaluation of the effects of agricultural management on biodiversity, showing uniform traits distribution when coping with the ecological factors that characterize the different plantation facets. We have found that carabid beetles can be used as model organisms for studying the effects of agricultural practices. Our study suggests that the interaction of man-induced trasformation with the natural background of the olive agroecosystem may be difficult to disentangle, so that such complexity must be taken into account when carabid beetles are expected to provide an ecosystem service for good agricultural practices.

## Introduction

Olive tree (*Olea europaea* L.) is one of the emblematic plants of the Mediterranean landscape, where it features an important role either cultural and economic. Therefore, the prevention of plant diseases and the maintenance of fruit production are of great concern.

The olive grove agroecosystem is an agricultural system designed and managed by man where the soil is periodically modified by agronomic practices, and the plants undergo chemical pesticide treatments to control outbreaks of pests, among which one of the most important is the olive fly [1–4], *Bactrocera (Daculus) oleae* (Gmelin, 1790) (Diptera: Tephritidae). Agronomic practices may have a negative impact on soil properties and erosion [5], while the consequence of the intensive use of pesticides often generates environmental modifications, which extensively impact on biodiversity [6–9]. Conventional agricultural practices affect the soil’s fauna diversity and activity by tillage and removing of the herbaceous vegetation cover [7, 10], the same is likely to happen as an effect of soil cultivation in olive groves. The impact of these management practices may cause a decrease in the number of epigean insects, as well as changes in their abundance [11, 12], leading to a detrimental effect even to beneficial insects (e.g. pest predators).

In recent times, the need for low-input agronomic techniques has encouraged the maintenance of agroecosystems entirely covered with spontaneous herbaceous vegetation (i.e. cover-cropping) [13, 14]. Monitoring the effects of agricultural practices by means of organisms sensitive to such kind of impact is a useful way for approaching the complexity of a comprehensive biodiversity study even over a small agroecosystem [15–18].

Although functional diversity (i.e., ‘the value and range of those features in species and organisms which influence how an ecosystem works’ [19]) is a key biodiversity parameter, studies focused on functional biodiversity in olive agroecosystems and data about the impact of cover crop on biodiversity in agricultural ecosystems are scarce [20], while quantification methods are still underdeveloped [20–22]. Notably, the classification of arthropod species strictly characterising the olive grove agroecosystem is still missing.

Carabid beetles have been proposed as beneficial insects in agroecosystems [23], or even as possible predators of *Bactrocera* [3, 4, 24, 25], and they are under risk if chemical or mechanical treatments alter the natural condition of the soil.

Carabids are epigean beetles living within the soil’s top horizons where they occupy several ecological niches and are a crucial component of predator diversity either in natural and in agricultural ecosystems [26, 27]. They respond to ecosystem alterations either at fine and at broad environmental scale [28, 29]. Different species show different feeding strategies, such as zoophagy (predatory), phytophagy (seed-eating) and polyphagy (zoophytophagous). Zoophagous types have a special role in containing phytophagous insects of agrarian significance, especially if the latter spend part of their biological cycle in the soil or within the herbaceous layer [11, 23, 30, 31]. Carabid assemblages are distributed in the environment according to definite species’ habitat preferences, which is also affected by landscape managements [11, 23, 32–34].

Even if the role of carabid beetles as indicators is controversial (see e.g. [35, 36]), it has been found that at least in the agricultural landscape they respond to the human artificialization of the environment [10, 16, 23], so that they can be used as model organisms exemplifying the insects living on the ground [18, 37, 38]. In particular, the main environmental factors affecting the species distribution are: the soil structure [23, 39], the type of soil management [40, 41] and the use of pesticides [9, 42]. This paper aims to study carabid beetles distribution in olive groves, for disentangling the possible consequences of different types of soil management. Given that there is a lack of knowledge about the relationships between agroecosystem and carabid communities, the latter need to be assessed in the Mediterranean agricultural landscape, thus the first question is: i) is species distribution affected by increased frequency of soil cultivation?; then ii) what are the main species characterizing olive plantations?; and iii) which species traits are positively/negatively selected by soil cultivation?

## Materials and methods

### Study area

**Fig 1:**
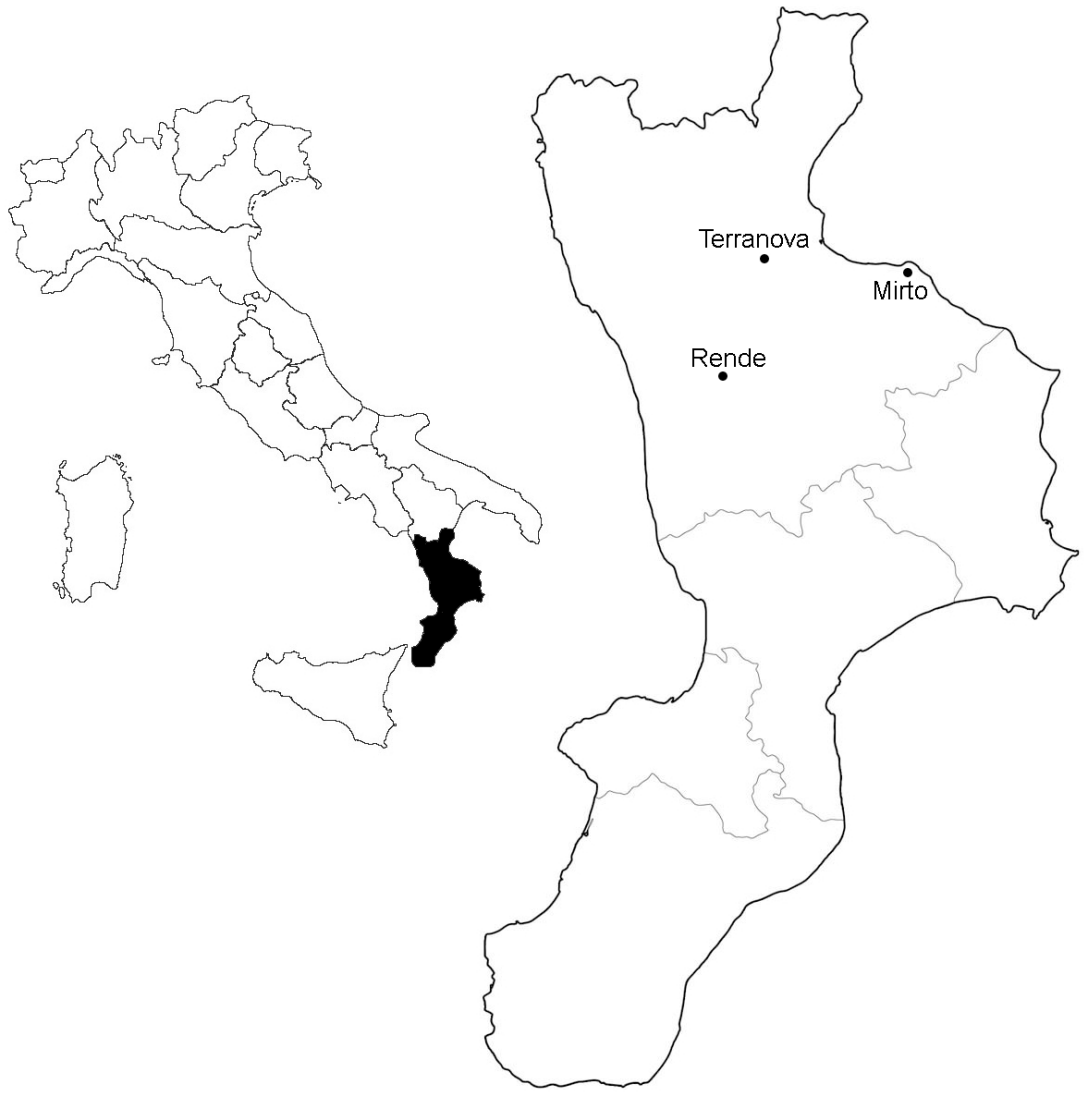
Geographical location of the sampled olive plantations. The Calabria study area, one of the southernmost regions of Italy, and in a central position within the Mediterranean basin. The REN sites were sampled in Rende, while the TER and MIR in Terranova and Mirto respectively.

The study area was in Calabria, one of the southernmost regions of Italy, and in a central position inside the Mediterranean basin. The olive plantations were located in the municipality of Rende (UTM 33S 606004.62 E; 4358146.54 N), Terranova da Sibari (UTM 33S 618990.68 E; 4392167.20 N) and of Mirto (UTM 33S 651740.77 E; 4386733.68 N). All the three study areas belong to the Cosenza Province, Italy (see Fig 1 and Table 1).

**Table 1:** Sample sites features.

Code	Management	m a.s.l.	Municipality	Soil	Forest climax
REN1ti	tilled	300	Rende	Sand/clay	Quercus virgiliana
REN2ti	tilled	300	Rende	Sand/clay	Quercus virgiliana
REN3ti	tilled	300	Rende	Sand/clay	Quercus virgiliana
REN4ti	tilled	300	Rende	Sand/clay	Quercus virgiliana
REN5cc	cover cropped	300	Rende	Sand/clay	Quercus virgiliana
REN6cc	cover cropped	206	Rende	Sand/clay	Quercus virgiliana
REN7cc	cover cropped	210	Rende	Sand/clay	Quercus virgiliana
TER1hc	half-cropland	75	Terranova	Clay	Mediterranean evergreen
TER2hc	half-cropland	73	Terranova	Clay	Mediterranean evergreen
TER3hc	half-cropland	71	Terranova	Clay	Mediterranean evergreen
TER4hc	half-cropland	60	Terranova	Clay	Mediterranean evergreen
TER5hc	half-cropland	50	Terranova	Clay	Mediterranean evergreen
TER6hc	half-cropland	45	Terranova	Clay	Mediterranean evergreen
TER7cc	cover cropped	170	Terranova	Sand/clay	Mediterranean evergreen
MIR1cc	cover cropped	5	Mirto	Alluvial	Lowland mixed
MIR2cc	cover cropped	5	Mirto	Alluvial	Lowland mixed
MIR4cc	cover cropped	5	Mirto	Alluvial	Lowland mixed

The code is composed by the first three letters of the municipality (i.e. direct intuitive geographically based classification), sequentially numbered, and followed by the management code. Management: tilled (ti) = periodically undergoing mechanical soil tillage; half-cropland (hc) = characterized by cover crop of spontaneous herbaceous vegetation only under tree rows, being the rest of the soil tilled; cover cropped (cc) = entirely covered with spontaneous herbaceous vegetation mowed in spring. Forest climax: the forest vegetation potentially growing in natural conditions (i.e. absence of agricultural landscape).

We compared ground beetle communities sampled from olive plantations, characterized by three types of soil management (i.e. the treatment) (Table 1). In the following the site code comes from the territorial location name of olive plantations (i.e. REN from Rende, TER from Terranova di Sibari and MIR from Mirto), so that a direct geographically based classification is intuitively given. Sample sites were (i) those periodically undergoing mechanical soil tillage (which, in the following, are called tilled sites with the code REN1-REN4; (ii) those characterized by cover crop of spontaneous herbaceous vegetation only under tree rows, with the rest of the soil tilled (half-cropland sites TER1-TER6); and (iii) those counted as no-treatment sites because entirely covered with spontaneous herbaceous vegetation, even if mowed in spring (cover-cropped sites REN5, REN6, REN7, TER7, MIR1, MIR2 and MIR4).

Each site was also characterised on the basis of the following factors: regional location, altitude, soil type, and potential natural vegetation (i.e. climax; see Table 1).

The pristine ecological landscape has been changed over the centuries by the spreading of agricultural practices, which lead to the present agricultural landscape. The natural vegetation substituted by olive plantations (i.e., the potential vegetation; see Table 1) belongs in the evergreen (mainly *Quercus ilex*) Mediterranean forest (TER sites), the sub-Mediterranean (*Quercus virgiliana*) deciduous forest (REN sites) and the lowland mixed forest (*Quercus robur*, *Fraxinus oxycarpa*; MIR sites). The soil of all sampled sites was a mixture of sand and clay, while only MIR sites grow on finer, silt-rich alluvium.

### Species traits

For each site the weight of each trait has been used for adding more information to the species distribution analysis, by analysing whether it is possible to link the dominance of one or more traits to agricultural practices.

Among the most frequently used morpho-functional traits [43, 43–46] we analysed body size, food choice and wing status (i.e. three morpho-functional traits), a biogeographical trait, i.e. the type of distribution range (chorotype: see [47] and habitat association and reproduction rhythm (i.e. two eco-physiological characteristics). The body size of each species has been measured on 20 individuals of both sexes, while other traits have been extracted from a database available from the Department of Biologia Ecologia et Scientiae Terrarum in the University of Calabria, as well as from recent literature (e.g., [48]). The species’ chorotypes were identified according to [49], after which they were grouped according to their range size (see also: [50]).

See S1 Table for the traits of each species.

### Quantitative data

Ground beetles were collected by using pitfall traps, which were emptied every 20 days, from April to December in 2005, in order to obtain a “year’s sample”. In this way the different seasonal period of activity of each species was intercepted by the traps, so avoiding the possible bias affecting the samples (as in [28, 51]; see also [39]). This is a standardized sampling method successfully applied since many years by many authors (see [28] and literature therein).

The traps were plastic vessels with an upper diameter of 9.2 cm, a depth of 11 cm and a small opening at 4 cm below the border, to avoid rainwater overflowing, filled with 200ml of a conservative mixture of wine vinegar and 5% ascorbic acid. In all sites, three traps were set up in a line at a distance of 15 m from each other. In total, 51 traps were utilized.

Quantitative data from traps were evaluated as annual Activity Density (aAD), i.e., as the number of individuals per trap per 10 trapping days for each species (see S1 Info aAD computation, for details on computation).

Pitfall traps intercept the activity of carabids, and the activity mirrors the way by which the species disperse and eventually cluster toghether, so that it is possible to describe the species groupings characterizing different habitats on the basis of their activity pattern. This was acknowledged in the traditional literature on carabid synecology (see the review in [52]), and by several authors working on carabid assemblages with an "abundance approach", as e.g. [28, 53–57].

Data have been analysed in R environment [58]. The variation of the species richness (i.e., number of species) and the activity (i.e. aAD) have been evaluated for the sampled sites by means of bootstrapping (“rich” function in [59]) to compare the actual number of sampled species (i.e. that species whose activity was intercepted by the traps) with the expected one (i.e. the bootstrapped one) to evaluate the eventual amount of lacking data.

On the basis of quantitative data (i.e., aAD), species have been classified by means of correlation coefficient and average linkage clustering algorithm, while sample sites have been classified by means of Bray-Curtis coefficient and minimum variance clustering algorithm. Principal Component Analysis (PCA) has been used to identify the main factors influencing the distribution of species over different habitats. PCA depicts components as orthogonal axes, and the relative position of sites on these axes was chosen as a tracer for each component (see [60]).

Given the high aAD variability of each carabid species among the sample sites, two threshold levels (‘‘central” and ‘‘nuclear”) were introduced with the exclusive intent of facilitating the interpretation of the data table (S1 Table; see [47]), i.e. a species is central in the site where it shows the maximum aAD, while it is nuclear in the site(s) where it shows an aAD equal to, or greater than, the mean aAD among the sites [61].

For each site the weight of each trait was computed as the number of species showing that trait, and this weight was used to add more information to the discussion of the results from the above analyses.

### Field permit

This study was carried out on private lands (Terranova and part of the Arcavacata olive groves) with the permission of the owners, and on public lands explicitly devoted to agricultural researches (Mirto and part of the Arcavacata olive groves) managed by the Council for Agricultural Research and Analysis of Agricultural Economics, Research Centre for Olive, Citrus and Tree Fruit. Field studies did not involved endangered or protected species.

## Results

The observed species richness (62 species) and mean activity density (aAD = 13.9) are within confidence intervals for the expected values computed by means of bootstrapping (61–79, 9.2–18.8, respectively); hence, sampling intensities were adequate in the studied sites. We collected 10,189 individuals belonging to 62 species, of which 32 were in tilled olive groves, 21 were in half-cropland and 49 were in cover-cropped fields. The aAD of the collected species, their scientific names and authorities, has been presented in S1 Table, which shows that most of the species were scattered among the different sample sites, with 31% of the species captured in one site (singletons) and 21% on two sites (doubletons). Only two generalist predators, *Pterostichus melas italicus* and *Calathus fuscipes*, were collected from all sites, with values ranging between 0.56–9.89 and between 0.71–31.92 respectively. Five species, *Pseudophonus rufipes*, *Calathus cinctus*, *Calathus montivagus*, *Carabus coriaceus mediterraneus* and *Laemostenus cimmerius*, were found in more than 50% of the sampled sites.

**Fig 2:**
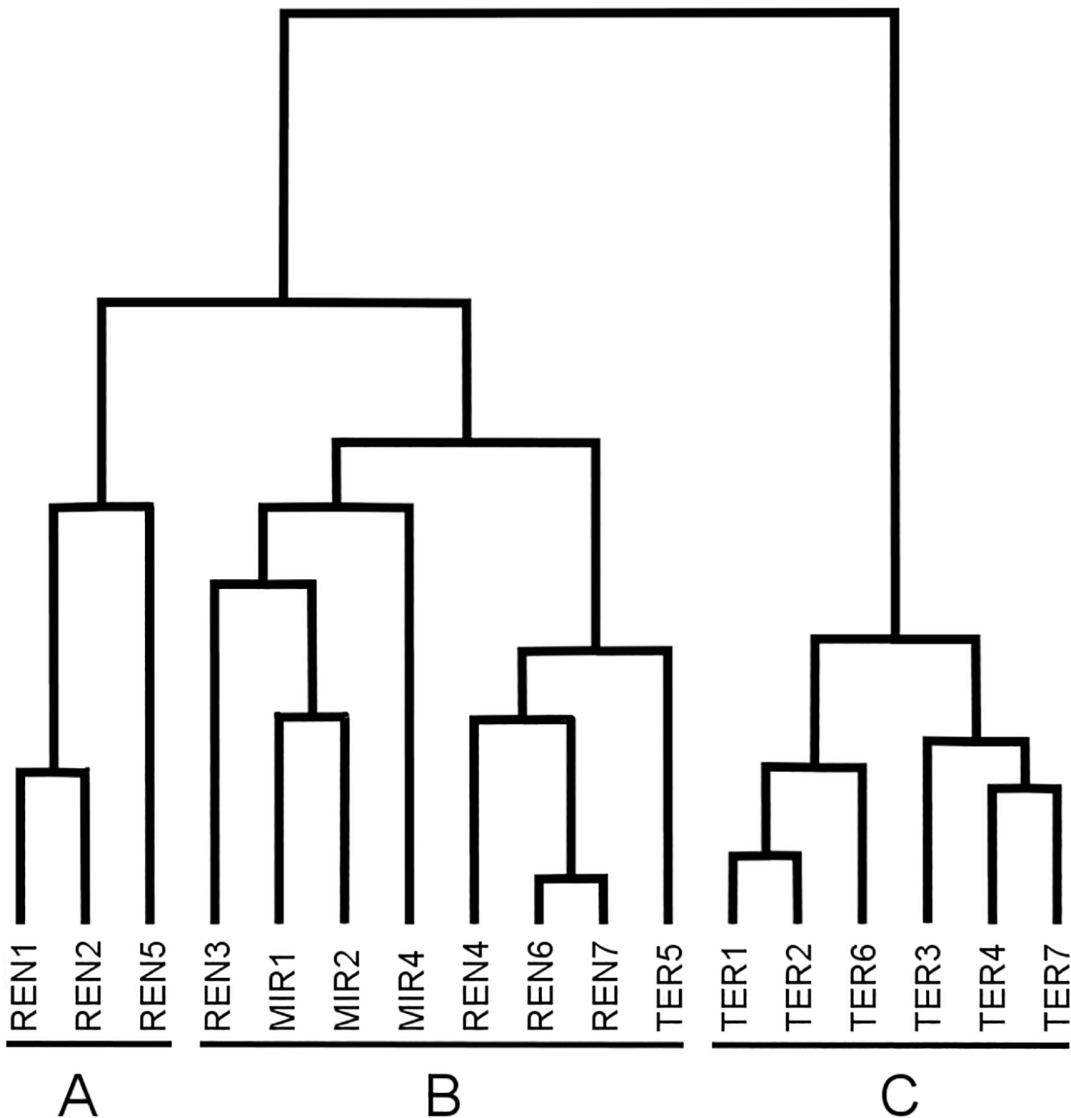
Sample sites classification by means of Bray-Curtis coefficient and minimum variance clustering algorithm. Three groups have been outlined and identified as A, B, C. REN sites belong to the Rende, TER to the Terranova and MIR to the Mirto municipalities respectively.

**Fig 3:**
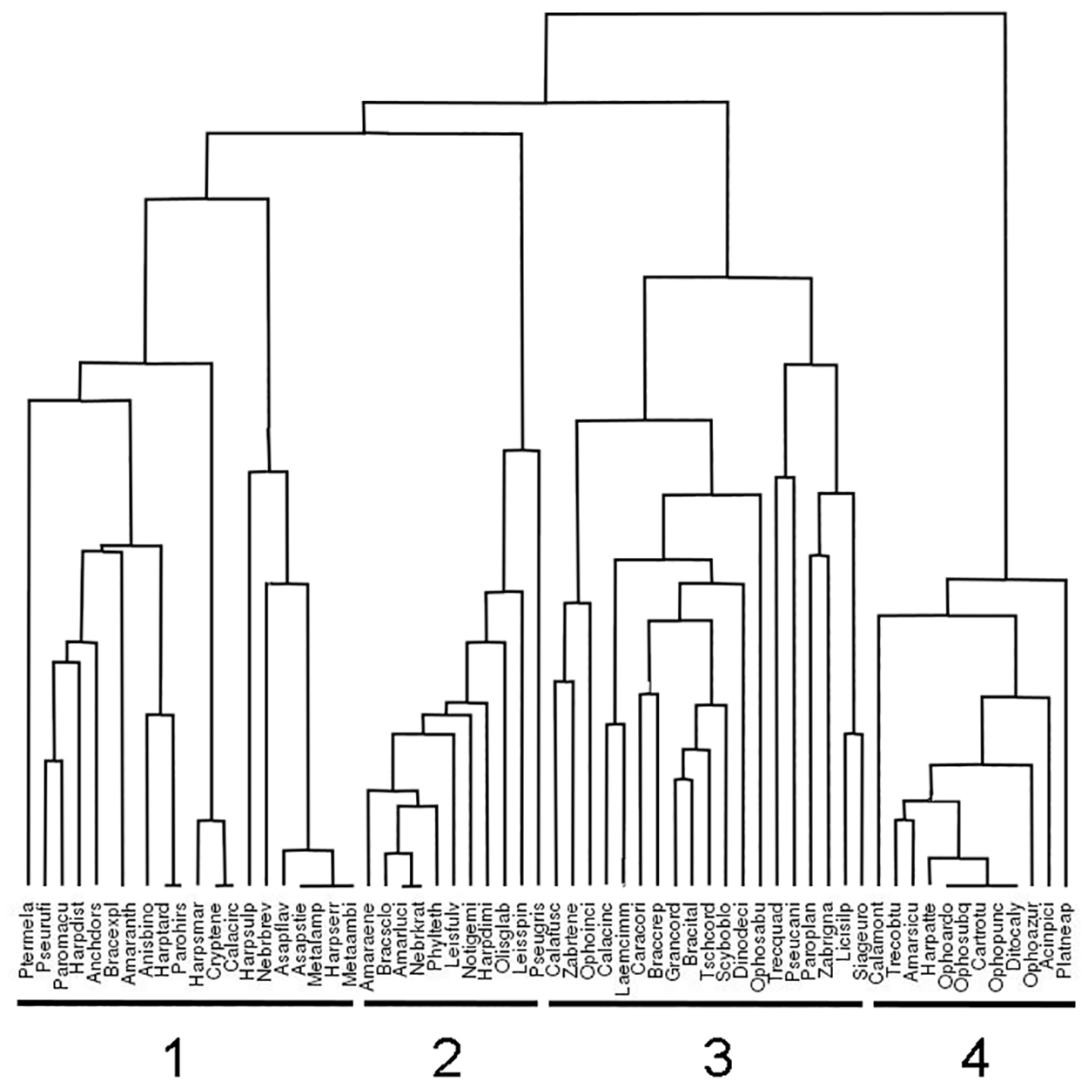
Species classification by means of correlation coefficient and average linkage clustering algorithm. Species have been classified into four groups (1 to 4 in the figure). The name of the species has been coded by merging the first four letters of the genus with the first four letters of the species name (see S1 Table).

The result of cluster analysis was the cross-classification of sites and species given in S1 Table, where the groups of sites A, B and C (Fig 2) and the groups of species 1, 2, 3, and 4 (Fig 3) were recognized. The similarity structure of the data was improved by highlighting central species in bold and nuclear species underlined. The sites belonging to groups A (three sites) and B (eight sites) are in the region of the deciduous forests, while the sites belonging to group C (six sites) are in the region of evergreen Mediterranean forests.

In the cross-classification of the group of sites A (Fig 2) with the group of species 4 (Fig 3) most of the species either have a central value in the sites of group A, showing no nuclear values in other sites, or were captured exclusively in such sites (see S1 Table). This is the only group in which the seed eater species are present in large numbers (24%), mainly tied to the REN5 site, which is characterized by cover-cropping. In the sites of group A there are scattered species automatically assigned (i.e., from the algorithm) to group 3 while reasonably belonging to group 4 (see S1 Table)

The largest group of sites is B, whose sites are located in different geographic districts, i.e., REN sites are located in the western part of the region, while MIR and TER are located in the eastern part. Most of the species captured in the sites of the B group belong to groups 1 and 2 (clustered into the same supergroup; see Fig 3). However, they also show central values (as well as only a few nuclear values) mainly in the REN3, MIR1, MIR2 and MIR4 sites, showing a structure of similarity that is more homogeneous by subclustering into a subgroup (see Fig 2), as distinct from the REN4, REN6-7 and TER5 sites, where the species are scattered among the B group sites.

In the cross-classification of the group of sites C with the group of species 3, the captured species were shared among sites, showing both central and nuclear values and belonging almost exclusively to the group of species 3 (Fig 3).

For analysing whether it was possible to characterize the clusters of sites on the basis of the dominances of the species traits, Table 2 was evaluated. It shows that the species traits were quite unevenly distributed among the several categories they were organized in. The most frequent body size was that of small species (i.e., size between 5mm and 10mm, s2 category in Table 2) within the whole sampled carabid fauna, where they accounted for the 53% of the species. The same s2 category was also the most frequent one in the group of sites A and B (53% and 51% respectively), while the s3 category was the most frequent (40%) in group C.

**Table 2:** Frequencies of the species traits.

Species trait Categories of trait	Code of the category	Species (total)	average cluster A	average cluster B	average cluster C
**Body Size (length)**					
≤ 5 mm	s1	10	13	5	0
> 5—≤ 10	s2	53	53	51	26
> 10—≤ 15	s3	24	17	29	40
> 15—≤ 20	s4	11	11	11	26
> 20—≤ 25	s5	0	0	0	0
> 25—≤ 30	s6	0	0	0	0
> 30 mm	s7	2	5	4	9
**Food of the adult**					
Specialist predators	Spc	8	8	8	4
Generalist predators	G	39	53	53	63
Phytophagous	P	16	8	2	6
Omnivorus (polyphagous)	Om	37	30	37	27
**Wing morphology**					
brachypterous	b	16	37	30	47
macropterous + dimorphic	m	84	63	70	53
**Chorology**					
Italian endemics	II	10	23	11	21
Euro-Mediterranean	IIIm	35	32	26	24
European	III	16	14	23	23
Eurasiatic/Eurosiberian	IV	21	12	14	11
Paleartic	V	18	19	25	22
**Habitat preference**					
Forest	F	20	37	23	23
Open habitat	O	66	45	54	55
Eurytopic	E	15	18	22	21
**Reproduction rhythm**					
Spring breeder	S	56	19	42	27
Autumn breeder	A	44	75	53	73

First column: in bold, the type of trait, under which, indented and in plain text, follows the list of the evaluated traits. Code: the code given to each species trait. For each trait the percentage of species bearing that trait was given on the basis of all sampled species in column "Species (total)", while the average percentage has been given for each group of sites, outlined by the cluster analysis of Fig 2, in columns "average A, B and C".

Among the four feeding strategies evaluated in Table 2, generalist predators were the most frequent species both within the whole sampled carabids (category G = 39%) and within each group of sites (category G never less than 53%), while polyphagy was the second most frequent feeding strategy.

Brachypterous species were the less frequent ones (always less than 53%) both among all the sampled species and within each group of sites.

The habitat preference of most of the species was for open habitats.

The most frequent reproduction rhythm among the whole sampled carabid fauna was given by the spring breeders, which instead were scattered among the three groups of sites, as autumn breeders were clearly the most frequent ones within each group.

The only exception was chorology (i.e., distribution range), where species were quite evenly distributed among the categories II to V as shown in Table 2.

Both the whole sampled carabid fauna and the sets of species within the three group of sites (A, B and C, Fig 2) can be considered as similar for what concerns the most frequent species traits, because all of them were characterized by the dominance of small species, macropterous species, generalist predators and autumn breeders, while they were quite homogeneous in respect to geographic distribution, as evaluated in Table 2.

To outline the species mainly related to olive plantations, among the several candidates, on the basis of their aAD, we highlighted that species with aAD values higher than average within each cluster of sites (see Fig 2 and S1 Table). After excluding the two forest species, i.e., *Calathus montivagus* and *Pterostichus melas*, it was possible to recognize 11 species, listed in Table 3, as characteristic of olive plantations. They showed a distribution range mainly around the Mediterranean region, and they are chiefly small species with a high dispersal power and an opportunistic diet (see Table 3).

**Table 3:** Carabid species characterizing olive plantations. The species showing aAD values above the average aAD within each group of sites outlined by cluster analysis have been listed.

species name	Body Size	Food of the adult	Wing morphology	Chorology	Habitat preference	Reproduction rhythm
Amara aenea	s2	Om	m	V	O	S
Brachinus sclopeta	s2	G	m	IIIm	O	S
Calathus cinctus	s2	G	b	V	O	A
Calathus fuscipes	s3	G	d	IIIm	E	A
Carterus rotundicollis	s2	P	m	IIIm	O	S
Harpalus attenuatus	s2	Om	m	IIIm	O	S
Harpalus smaragdinus	s2	Om	m	IV	O	S
Ophonus ardosiacus	s3	P	m	IIIm	O	A
Ophonus azureus	s2	Om	d	IV	O	n.a.
Pseudophonus rufipes	s3	Om	m	V	E	A
Scybalicus oblongiusculus	s3	Om	m	IIIm	O	S

The meaning of the codes under the columns different from "species name" is the same as in Table 2.

The results of PCA showed that it is not possible to highlight one single factor (i.e. axis) mainly affecting data ordination, because 20% of data variability is distributed along the first axis of the space outlined by the principal components, while 18% and 14% is distributed along the second and third axes respectively, i.e., only half of the entire variability is explained by means of the three most meaningful principal components. Moreover, the sample sites were drawn close together and mainly around the axes’ origin, which is a probable symptom of the lack of preponderance among the several factors affecting species distribution.

**Fig 4:**
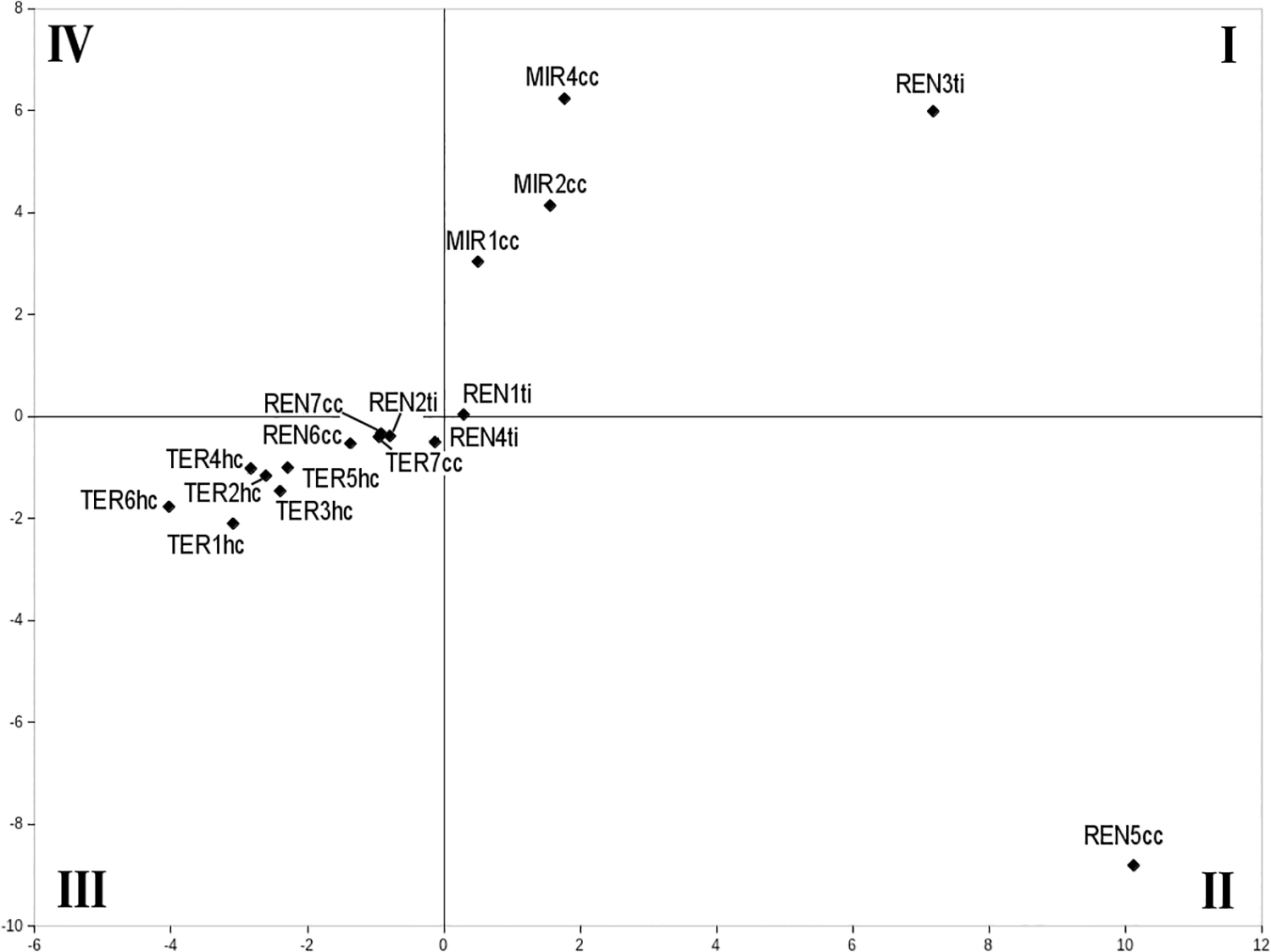
PCA ordination of the sample sites. The space has been identified in four quadrants (I—IV) to facilitate the interpretation of sites position. REN and LIR sites belong to the Rende, TER to the Terranova and MIR to the Mirto municipalities respectively.

The ordination of the sites along the first axis (Fig 4, S2 Table) mirrors the potential vegetation characterizing them, even though some exceptions are evident (i.e., REN6 and REN7 sites). On the left part of the axis, a set of sites characterized by evergreen Mediterranean vegetation has been projected, while the deciduous forest sites have been projected on the right.

Along the second axis (Fig 4, S2 Table), the order of the sites mirrors the soil type. Going from down to top, it is possible to recognize a sequence from clay through sand clay to alluvial soil, i.e., from dry to damp soils, although sites REN5 and REN3 do not follow such ordination.

The soil management is probably tied to the third axis (S2 Table), where it is possible to see a separation between the sites affected by man-originated disturbance (negative side of the third axis) and the sites characterized by cover-cropping (positive side), with the exception of REN2.

**Fig 5:**
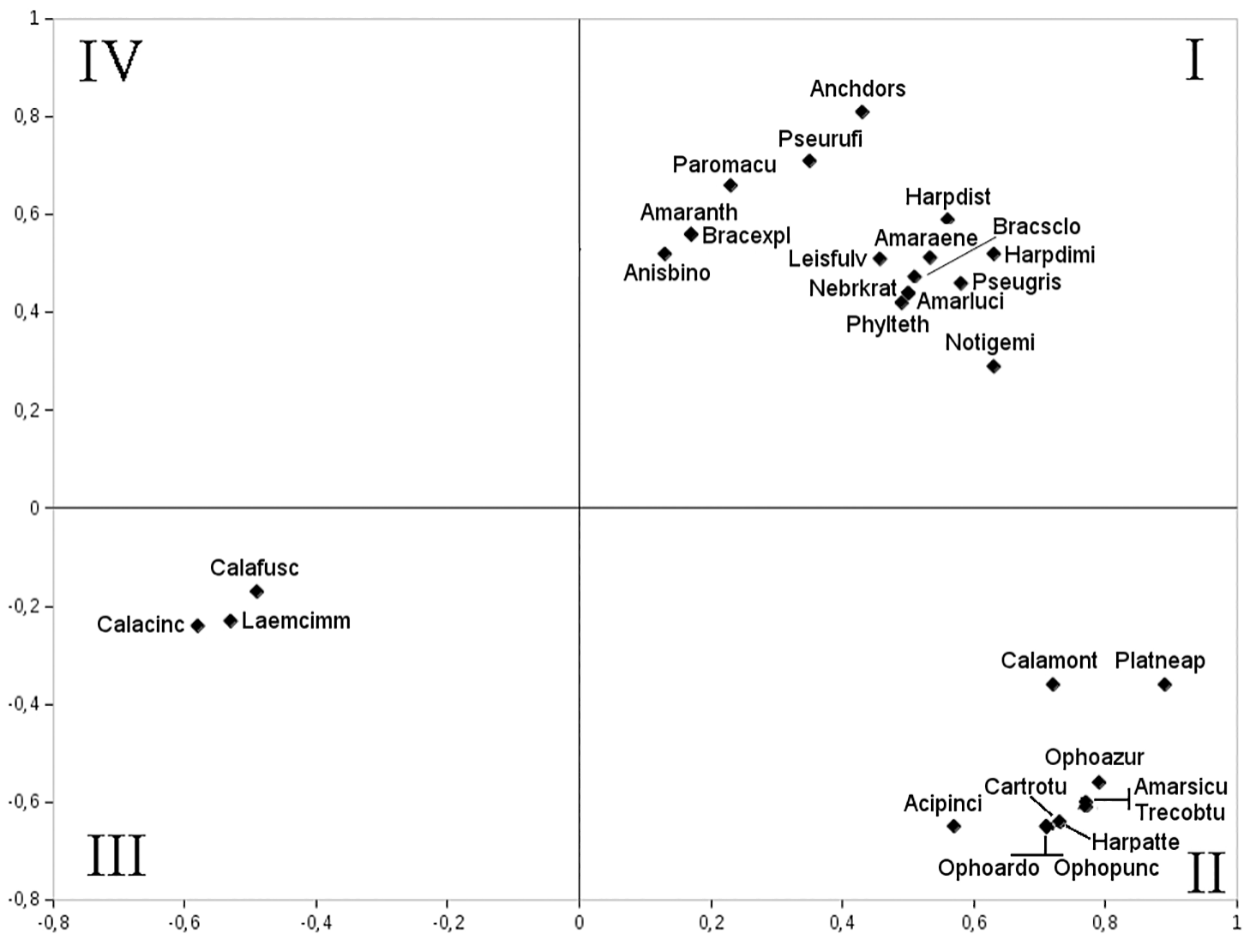
PCA ordination of the species, showing the species with the highest meaningful correlation with the first two axes. The space has been identified in four quadrants (I—IV) to facilitate the interpretation of species vs. sites position. The name of the species has been coded by merging the first four letters of the genus with the first four letters of the species name (see S1 Table for species names).

The ordination of our data showed that (see Fig 4, quadrant II of the space generated by the PCA) in olive plantations that grow within a potential deciduous oak region over dry soils, and in which disturbance generated by man was limited to seasonal grass mowing, feeding strategies that exploit vegetal resources in an opportunistic (e.g., *Amara sicula*, *Harpalus attenuates* and *Ophonus azureus*; see Fig 5) or exclusive way (i.e., seed-eating species, such as *Carterus rotundicollis* and *Ophonus ardosiacus*) were positively selected. Meanwhile, when under the same potential vegetation, humid soil conditions, the disturbance by man is greater (PCA quadrant I; Fig 4), then seed eaters left room for opportunistic or predator feeding strategies (e.g., *Anchomenus dorsalis*, *Pseudophonus rufipes* and *Brachinus sclopeta*; see Fig 5).

It is likely that the life strategies which were successful to survive in deciduous forest conditions, were positively selected for the bioclimatic Mediterranean belt too (PCA quadrant III; Fig 4), but only to some extent. Carabid populations colonizing olive plantations of the Mediterranean belt were characterized almost entirely by generalist zoophagous predators (e.g., *Calathus fuscipes*) and the lack of specialized predators, while the latter were present with a few species in deciduous forests. They were also characterized by a chorological spectrum dominated by Mediterranean species; and by low body size variability, mainly restricted to s3 category (a category size of 10-15mm).

## Discussion

Olive cultivation in Calabria is characterized by highly heterogeneous agricultural management practices, often affected by the interaction of microclimatic and soil features. Olive plantations were introduced in the hill landscape (49%) and in montane areas (42%) mainly, while only 9% were introduced in the lowlands [62], over a wide range of soil types, aspect, tillage type and potential climax forestation.

The distribution of carabid species living in the studied agricultural landscape is characterized by the high number of singletons (31%) and doubletons (21%), which account for more than half of the sampled fauna. The species distribution is probably due to the complexity of the interaction between semi-natural and artificial (i.e., man-originated) environmental factors.

On the basis of the classification, it was not possible to outline a clear species vs. site cluster structure. Instead it was found an evident trend of site-by-site species clustering, which was probably affected by the ecological factors characterizing every single sample site. This is the case, e.g., of the sites belonging to the A group, where the fact of belonging to the same geographical district may have influenced species distribution, while in the group C the natural and man-made disturbance is likely to be more critical than sharing the same geographical district.

After the classification of the sites, it was possible to characterize the groups of the sites on the basis of the potential vegetation they were located within, while other factors recorded in the sites did not mirror site clustering (see Table 1 and Fig 2).

Ordination showed that at least three factors affected the species distribution among the sampled sites, namely (in an apparent order of importance), potential vegetation, soil type and soil management, so that the outlining of a clear ecological gradient is probably hindered by the interaction among such factors. On the basis of the explained variability by the first three PCA axes (i.e., 50%), and the indirect gradient analysis arguable by the site distribution along the axes, it is indeed possible to hypothesize that, in our sites, each factor was slightly prevailing on the others at the local scale in an apparent random manner. Similar results have been found by [63] where soil management was one among several factors affecting arthropods distribution in olive agroecosystem, and by [64], where groundcover vegetation played different roles at different geographical scales. It is not to be excluded that even in the studied sites the interaction of several factors, e.g. soil tillage, residue management and N fertilization rate, could affect soil biota diversity as found by [65].

An answer to our first question comes from the olive grove picture emerging from our work, and from the literature, which is that of an agroecosystem showing an uniform physiognomy, under which several local factors, either natural and man-induced, exerted a driving influence on the local composition of species assemblages. Different life strategies (i.e., species traits) eased to cope with the local driving factors, as Table 2 showed, but we found that each species trait was equally dominant in the sampled sites even though soil cultivation techniques were different.

The above confirms that the olive grove is a complex agroecological system, where natural resources, like carabids, are not linearly affected by the intensity of agricultural practices, i.e. soil cultivation technique is not the preponderant factor affecting specie distribution.

The second question of our paper is to be framed into an historical perspective. The sampled sites belonged to the same ecological landscape, and their agricultural dimension was developed by the same agricultural practices, so that they represent a sort of homogeneous unity, both under present anthropic factors and historical landscape modification point of view.

On the basis of the data heterogeneity and landscape features outlined above, we interpret the carabid distribution in olive plantations as stemming from two sets of species, one set originating from forest and the other from non-forest ecosystems. The two sets actually coexist in the studied sites, giving rise to several species groupings, depending on the ecological factors prevailing within each olive plot. A set of a few species originating from natural forest ecosystems (e.g., *Pterostichus melas*, *Calathus montivagus*, *Laemostenus cimmerius*) was able to resist man-induced land modifications (i.e., agriculture) by keeping viable populations in olive plantations. This was also thanks to a probable turnover from the surrounding forest environments, as observed for Lepidoptera [66]. A set of species originating from non-forest ecosystems found in olive plantations a new environment, which was to be colonized (e.g., *Calathus fuscipes* and *Pseudophonus rufipes*), to be opportunistically exploited (e.g., *Amara aenea*, *Brachinus sclopeta*, *B*. *italicus* and *Scybalicus oblongiusculus*), or facilitated fitting microhabitat conditions (e.g., soil humidity for Bembidiinae).

It is also not to be excluded, and it should be further investigated, that the presence of higher-order interactions (HOIs in [67]) among carabid populations lead to the local formation of species groupings. Such local species groupings, filtered by the local diversity of the abiotic factors, actually would be facies of a higher level community, i.e. a sort of super-community.

Question ii) posed in the introduction remains partly open, because on the basis of cluster analysis and PCA, it is likely that many species characterized the different facets of olive plantation. Since we observed several species showing exclusive preference for only one of the several olive plantation facets, it was not possible to list a group of species as indicators of the olive agroecosystem, while it was possible to outline in Table 3 the species mainly related to the sampled olive groves.

This suggests that throughout the Mediterranean region it will be likely to find different species groups in relation to geographical factors (e.g., compare the results of [4]). Our third question concerns the relationship between the faunistic geographical variation by analysing the life strategies positively or negatively affected by soil cultivation.

The interpretation of carabid distribution on the basis of species traits (i.e., life strategies) shows that, after evident species diversity, uniform life strategies are dominant when the species had to cope with the ecological factors that characterize the different plantation facets.

The general dominance of autumn breeders is due to mowing and tillage, which are practised in spring, negatively selecting those species laying eggs in spring. It is possible to hypothesize that, as a rule within the agricultural landscape, the positive selection of a given seasonal breeding strategy mirrors seasonal soil management.

Macropterous species dominate the complex of species groupings because olive plantation, although it is a permanent tree agroecosystem, it is far from being a semi-natural environment, at least in Calabria, where a remarkable amount of artificial disturbance, mainly at soil level, is always present, and against which high dispersal power offers an adequately resilient strategy.

Under a perspective based on ecological gradient analysis, we found that the principal factors driving species distribution were the potential vegetation and soil type. Within the region of potential oak forests, on damped soils the omnivorous or the seed eating species found better conditions than the zoophagous species, which instead found better conditions on dry soils. Within the region of the potential Mediterranean vegetation, feeding strategy is more uniformly characterised by generalist predators species, with no apparent influence from the soil type.

The results from both taxonomic and biological analyses are congruent with similar studies in agroecosystems [42] (and literature therein) and suggest that carabid beetles can be used as model organisms exemplifying the response of insects living on the ground of olive groves.

They may represent an ecosystem service for controlling pest insects that spend part of their biological cycle on the gro und. In particular, we suggest that *Pterostichus melas* and *Calathus fuscipes*, generalist predators and autumn breeders, could be potential natural resources against *Bactrocera*. *oleae* in autumn [68, 69]. Furthermore, they are the only species present in all the sampled sites showing wide ecological tolerance. The same role may be played by *Pseudophonus rufipes*, as demonstrated by [70] against *Ceratitis capitata*.

## Conclusion

Our work is the first attempt to describe carabid beetle communities in Mediterranean olive plantations on the basis of the classical approach of species-by-site sampling, which we have further improved by weighting the species traits (for other types of cultivation see [71–73]). We have demonstrated that an informative approach ought to take into account community parameters that are different from the richness and abundance of species, to highlight the effects of agricultural management on biodiversity. A combination of taxonomic and functional approaches provides extra information confirming that the analysis of species’ morpho-functional features is a promising and common approach to evaluate functional diversity [21, 47, 74].

Our work suggests that a functional approach can reveal the interactions between biocoenosis and the factors affecting agroecosystems, not only spatially but also temporally. Adequate agricultural management should stimulate the presence of ‘positive’ arthropods, which could be able to restrain pest population directly and/or indirectly in a natural fashion. Future research should be focused on the role of medium-sized carabids, autumn breeders, to controle the olive fly and possibly other crop pests. Moreover, as we have found that several factors affect almost to the same degree the species distribution, the complexity of the olive agroecosystem must be considered when planning to use carabids for providing ecosystem services.

## Supporting information

S1 Table Species by sites table. Sites and species have been clustered following the classification of Fig 2 (groups A, B and C) and 3 (groups 4, 2, 1and 3).
(PDF)

S2 Table PCA coordinates. Sample sites ordination on the basis of PCA axis 1 (Table 2.1), axis 2 (Table 2.2) and axis 3 (Table 2.3).
(PDF)

S1 Info aAD computation Computation of the annual Activity Density (aAD). (PDF)
