# Supplementary material for: Ground beetles in Mediterranean olive agroecosystems: Their significance and functional role as bioindicators (Coleoptera, Carabidae)
Source: PLoS One. 2018 Mar 20;13(3):e0194551. doi: 10.1371/journal.pone.0194551 (PMC5860759; doi:10.1371/journal.pone.0194551)
Supplement: S1 Table — Sites and species have been clustered following the classification of Fig 2 (groups A, B and C) and 3 (groups 4, 2, 1and 3). (PDF) [file pone.0194551.s001.pdf]

S1 Table. Species by sites table. Sites and species have been clustered following the classification of Figures 2 (groups A, B and C) and 3 (groups 4, 2, 1and 3).
The species traits have been coded in the first six columns (see table 2 in the text). The name of the species has been coded by merging the first four letters of the genus with the first four letters of the species name.
Figures in bold outline the central value, while figures underlined outline the nuclear value (see text under Methods chapter)

								clusters	**A**			**B**								**C**					
								masl	300	300	300	300	5	5	5	300	206	210	50	75	73	45	71	60	170
								management	cover-c	tilled	tilled	tilled	cover-c	cover-c	cover-c	tilled	cover-c	cover-c	half-c	half-c	half-c	half-c	half-c	half-c	cover-c
								soil	sand-clay	sand-clay	sand-clay	sand-clay	alluv	alluv	alluv	sand-clay	sand-clay	sand-clay	clay	clay	clay	clay	clay	clay	sand-clay
								climax	Q.virg	Q.virg	Q.virg	Q.virg	mix.for	mix.for	mix.for	Q.virg	Q.virg	Q.virg	sclerof	sclerof	sclerof	sclerof	sclerof	sclerof	sclerof
							**clusters**	sites	REN5	REN2	REN1	REN3	MIR1	MIR2	MIR4	REN4	REN6	REN7	TER5	TER1	TER2	TER6	TER3	TER4	TER7
**Repr**	**Chor**	**Wing**	**Size**	**Food**	**Habitat**	**species**																			
2Y	II	b	s2	G	F	Platyderus neapolitanus Binaghi in Magistretti 1955		Platneap	**0.048**			0.027													
S	IV	m	s4	Om	O	Acinopus picipes (Olivier 1795)		Acinpici	**0.048**														0.024		
U	IV	d	s2	Om	O	Ophonus azureus (F. 1775)		Ophoazur	**0.605**			0.137					0.076	0.028				0.025			
S	IV	m	s4	P	O	Ditomus calydonius calydonius (Rossi 1790)		Ditocaly	**0.169**																
A	IV	m	s2	P	O	Ophonus puncticeps Stephens 1828		Ophopunc	**0.169**																
S	IIIm	m	s2	P	O	Carterus rotundicollis Rambur 1837		Cartrotu	**0.242**																
A	IIIm	m	s2	P	O	Ophonus subquadratus (Dejean 1829)	**4**	Ophosubq	**0.194**																
A	IIIm	m	s3	P	O	Ophonus ardosiacus (Lutshnik 1922)		Ophoardo	**0.363**																
S	IIIm	m	s2	Om	O	Harpalus attenuatus Stephens 1828		Harpatte	**0.630**			0.027													
A	II	b	s2	Om	O	Amara sicula Dejean 1831		Amarsicu	**0.218**			0.027													0.025
A	IIIm	b	s1	G	F	Trechus obtusus lucanus Focarile 1949		Trecobtu	**0.218**	0.023	0.024	0.027				0.024									
A	II	b	s2	G	F	Calathus montivagus Dejean 1831		Calamont	**0.508**	0.023	0.049	0.082	0.196	0.174	0.106	0.266	0.025		0.045	0.101	0.022	0.050			0.074
A	V	m	s2	Om	O	Pseudophonus griseus (Panzer 1797)		Pseugris	0.048	0.023	0.122	**0.137**		0.131	0.064							0.025			
A	II	m	s2	Spc	F	Leistus spinibarbis fiorii Lutshnik 1913		Leisspin		0.023	0.122	**0.137**													
A	IIIm	m	s2	G	O	Olisthopus glabricollis (Germar 1817)		Olisglab				**0.027**				0.024									
S	III	m	s3	Om	O	Harpalus dimidiatus Dejean 1829		Harpdimi	0.048			**0.384**		0.044	0.106	0.024									
S	IIIm	m	s2	Spc	O	Notiophilus geminatus Dejean & Boisduval 1830		Notigemi	0.024			**0.110**						0.028							
A	III	m	s2	Spc	F?	Leistus fulvibarbis fulvibarbis Dejean 1826	**2**	Leisfulv				**0.110**	0.022	0.022								0.025			
S	IIIm	m	s1	G	O	Phyla tethys (Netolitzky 1926)		Phylteth				**0.082**				0.024									
A	III	b	s4	G	F	Nebria kratteri Dejean & Boisduval 1830		Nebrkrat				**0.027**													
S	V	m	s1	Om	O	Amara lucida (Duftschmid 1812)		Amarluci				**0.055**													
S	IIIm	m	s2	G	O	Brachinus sclopeta (F. 1792)		Bracsclo				**1.781**	0.065	0.065	0.021										0.148
S	V	m	s2	Om	O	Amara aenea (Degeer 1774)		Amaraene	0.048			**1.918**	0.174		0.276	0.121									0.346
S	IIIm	m	s1	G	O	Metallina ambigua (Dejean 1831)		Metaambi					**0.087**												
S	V	m	s3	Om	O	Harpalus serripes serripes (Quensel 1806)		Harpserr					**0.022**												
S	V	m	s1	G	E	Metallina lampros (Herbst 1784)		Metalamp					**0.022**												
S	IIIm	m	s2	G	E	Asaphidion stierlini (Heyden 1880)		Asapstie					**0.022**												
S	IV	m	s2	G	E	Asaphidion flavipes (L. 1761)		Asapflav					**0.326**		0.021										
A	IV	m	s2	G	E	Nebria brevicollis F. 1792		Nebrbrev					0.022				**0.025**								
S	IIIm	d	s2	Om	O	Harpalus sulphuripes sulphuripes Germar 1824		Harpsulp	0.048			0.164	**0.283**	0.152											
A	IIIm	m	s2	G	O	Calathus circumseptus Germar 1824		Calacirc						**0.022**											
A	V	m	s2	P	F	Cryptophonus tenebrosus (Dejean 1829)		Cryptene						**0.022**											
S	IV	m	s2	Om	O	Harpalus smaragdinus (Duftschmid 1812)	**1**	Harpsmar				0.027	0.174	**1.959**	0.149										
S	IV	m	s2	Om	O	Parophonus hirsutulus (Dejean 1829)		Parohirs							**0.021**										
S	V	m	s2	Om	F	Harpalus tardus (Panzer 1797)		Harptard							**0.021**										
S	III	m	s3	Om	E	Anisodactylus binotatus (F. 1787)		Anisbino					0.022		**0.064**										
S	III	m	s2	Om	F	Amara anthobia Villa & Villa 1833		Amaranth						**0.022**	0.021										
S	IV	m	s2	G	F	Brachinus explodens (Duftschmid 1812)		Bracexpl						**0.022**	0.021										
S	V	m	s2	Om	O	Anchomenus dorsalis (Pontoppidan 1763)		Anchdors				0.082	0.044	0.087	**0.106**										
S	III	m	s2	Om	O	Harpalus distinguendus (Duftschmid 1812)		Harpdist	0.169	0.023		0.247	0.196	0.261	**0.510**										0.148
S	III	m	s2	Om	O	Parophonus maculicornis (Duftschmid 1812)		Paromacu				0.027	0.065	0.044	**0.170**										
A	V	m	s3	Om	E	Pseudophonus rufipes (Degeer 1774)		Pseurufi	0.266	0.023		1.260	1.545	1.937	**3.885**	0.048	0.076	0.056	0.023	0.025		0.025	0.047	0.023	0.025
A	III	b	s4	G	F	Pterostichus melas italicus (Dejean 1828)		Ptermela	0.751	0.557	0.998	1.479	1.219	2.089	**9.894**	2.179	1.751	1.653	3.108	1.131	2.172	6.584	3.626	5.075	0.296
S	IV	m	s3	Spc	O	Siagona europaea Dejean 1826		Siageuro									**0.051**	0.028							
A	III	m	s3	Spc	O	Licinus silphoides (Rossi 1790)		Licisilp									**0.127**						0.047		0.025
A	IIIm	m	s4	Om	O	Zabrus ignavus ignavus Csiki 1907		Zabrigna																	**0.198**
S	IIIm	m	s2	Om	O	Parophonus planicollis (Dejean 1829)		Paroplan		**0.023**															
?	IIIm	m	s2	G	F	Pseudomasoreus canigoulensis (Fairmaire 1854)		Pseucani			**0.049**				0.021										
A	IV	m	s1	G	E	Trechus quadristriatus (Schrank 1781)		Trecquad	0.024		**0.049**					0.024									
A	IIIm	d	s3	G	E	Calathus fuscipes graecus Dejean 1831		Calafusc	0.460	0.719	0.706	1.918	10.076	4.701	0.786	1.380	2.817	2.717	4.212	12.688	13.871	11.347	**31.919**	19.562	17.975
A	IV	m	s4	Om	O	Zabrus tenebrioides tenebrioides (Goeze 1777)		Zabrtene												0.126		0.025	**0.166**	0.069	
A	IIIm	m	s3	P	O	Ophonus incisus (Dejean 1829)		Ophoinci															**0.024**		
A	V	b	s2	G	O	Calathus cinctus Motschulsky 1850	**3**	Calacinc	0.024	0.116		0.027	0.479	0.283		0.024	0.406	0.168	1.599	0.829	1.312	2.668	1.374	**2.930**	0.123
A	III	b	s4	G	F	Laemostenus cimmerius (Fischer-Waldheim 1823)		Laemcimm					0.087	0.022	0.085				0.090	0.704	0.581	0.623	0.545	**1.176**	0.198
A	II	b	s7	G	O	Carabus coriaceus mediterraneus Born 1906		Caracori	0.048		0.024	0.027				0.024	0.051		0.270	**0.930**	0.344	0.424	0.071	0.277	0.025
S	V	m	s2	G	O	Brachinus crepitans (L. 1758)		Braccrep				0.082							0.045	**0.302**	0.151	0.299			
S	IIIm	m	s3	P	O	Graniger cordicollis (Serville 1821)		Grancord														**0.050**			
S	II	b	s3	G	E	Brachinus italicus (Dejean 1831)		Bracital											0.158	0.226	0.172	**1.222**		0.115	
S	IIIm	m	s3	P	O	Tschitscherinellus cordatus (Dejean 1825)		Tschcord											0.023			**0.025**			
S	IIIm	m	s3	Om	O	Scybalicus oblongiusculus (Dejean 1829)		Scyboblo									0.025		0.833	0.628	1.204	**1.421**		0.138	
S	IIIm	m	s3	G	O	Dinodes decipiens (Dufour 1820)		Dinodeci								**0.048**			0.023	0.025	0.022	0.025			
A	IV	m	s3	P	O	Ophonus sabulicola (Panzer 1796)		Ophosabu	0.024											**0.075**		0.025			
								aAD	5.4	1.6	2.1	10.4	15.1	12.1	16.3	4.2	5.4	4.7	10.4	17.8	19.8	24.9	37.8	29.4	19.6
								n.species	25	10	9	28	21	19	20	13	11	7	12	13	10	18	10	9	13
									ARC5	ARC2	ARC1	ARC3	MIR1	MIR2	MIR4	ARC4	LIR1	LIR2	TER5	TER1	TER2	TER6	TER3	TER4	TER7
