# Supplementary material for: Ground beetles in Mediterranean olive agroecosystems: Their significance and functional role as bioindicators (Coleoptera, Carabidae)
Source: PLoS One. 2018 Mar 20;13(3):e0194551. doi: 10.1371/journal.pone.0194551 (PMC5860759; doi:10.1371/journal.pone.0194551)
Supplement: S2 Table — Sample sites ordination on the basis of PCA axis 1 (Table 2.1), axis 2 (Table 2.2) and axis 3 (Table 2.3). (PDF) [file pone.0194551.s002.pdf]

S2 Table. PCA coordinates. Sample sites ordination on the basis of PCA axis 1 (S2.1 Table), axis 2 (S2.2 Table) and axis 3 (S2.3 Table).

S2.1 Table

site	Axis 1	m asl	managment	soil	veg
TER6hc	-4,03	45	half-cropland	clay	sclerof
TER1hc	-3,09	75	half-cropland	clay	sclerof
TER4hc	-2,83	60	half-cropland	clay	sclerof
TER2hc	-2,61	73	half-cropland	clay	sclerof
TER3hc	-2,4	71	half-cropland	clay	sclerof
TER5hc	-2,29	50	half-cropland	clay	sclerof
REN6cc	-1,37	206	cover-cropped	sand-clay	Q.virg
TER7cc	-0,95	170	cover-cropped	sand-clay	sclerof
REN7cc	-0,92	210	cover-cropped	sand-clay	Q.virg
REN2ti	-0,79	300	tilled	sand-clay	Q.virg
REN4ti	-0,13	300	tilled	sand-clay	Q.virg
REN1ti	0,29	300	tilled	sand-clay	Q.virg
MIR1cc	0,5	5	cover-cropped	alluv	mix.for
MIR2cc	1,56	5	cover-cropped	alluv	mix.for
MIR4cc	1,77	5	cover-cropped	alluv	mix.for
REN3ti	7,18	300	tilled	sand-clay	Q.virg
REN5cc	10,12	300	cover-cropped	sand-clay	Q.virg

S2.2 Table

site	Axis 2	m asl	managment	soil	veg
REN5cc	-8,81	300	cover-cropped	sand-clay	Q.virg
TER1hc	-2,1	75	half-cropland	clay	Med. ev.
TER6hc	-1,77	45	half-cropland	clay	Med. ev.
TER3hc	-1,46	71	half-cropland	clay	Med. ev.
TER2hc	-1,16	73	half-cropland	clay	Med. ev.
TER4hc	-1,02	60	half-cropland	clay	Med. ev.
TER5hc	-1	50	half-cropland	clay	Med. ev.
REN6cc	-0,53	206	cover-cropped	sand-clay	Q.virg
REN4ti	-0,5	300	tilled	sand-clay	Q.virg
TER7cc	-0,4	170	cover-cropped	sand-clay	Med. ev.
REN2ti	-0,38	300	tilled	sand-clay	Q.virg
REN7cc	-0,33	210	cover-cropped	sand-clay	Q.virg
REN1ti	0,04	300	tilled	sand-clay	Q.virg
MIR1cc	3,04	5	cover-cropped	alluv	mix.for
MIR2cc	4,14	5	cover-cropped	alluv	mix.for
REN3ti	5,99	300	tilled	sand-clay	Q.virg
MIR4cc	6,24	5	cover-cropped	alluv	mix.for

S2.3 Table

site	Axis 3	m asl	managment	soil	veg
REN3ti	-8,36	300	tilled	sand-clay	Q.virg
TER6hc	-2,01	45	half-cropland	clay	Med. ev.
TER1hc	-1,4	75	half-cropland	clay	Med. ev.
REN4ti	-0,99	300	tilled	sand-clay	Q.virg
TER2hc	-0,85	73	half-cropland	clay	Med. ev.
TER5hc	-0,65	50	half-cropland	clay	Med. ev.
TER4hc	-0,46	60	half-cropland	clay	Med. ev.
REN1ti	-0,36	300	tilled	sand-clay	Q.virg
TER3hc	-0,21	71	half-cropland	clay	Med. ev.
REN7cc	-0,2	210	cover-cropped	sand-clay	Q.virg
TER7cc	-0,15	170	cover-cropped	sand-clay	Med. ev.
REN2ti	-0,1	300	tilled	sand-clay	Q.virg
REN6cc	0,34	206	cover-cropped	sand-clay	Q.virg
REN5cc	2,43	300	cover-cropped	sand-clay	Q.virg
MIR2cc	3,41	5	cover-cropped	alluv	mix.for
MIR1cc	3,64	5	cover-cropped	alluv	mix.for
MIR4cc	5,92	5	cover-cropped	alluv	mix.for
