# Supplementary material for: Ground beetles in Mediterranean olive agroecosystems: Their significance and functional role as bioindicators (Coleoptera, Carabidae)
Source: PLoS One. 2018 Mar 20;13(3):e0194551. doi: 10.1371/journal.pone.0194551 (PMC5860759; doi:10.1371/journal.pone.0194551)
Supplement: S1 Info aAD computation — (PDF) [file pone.0194551.s003.pdf]

**S1 Info. Computation of the annual Activity Density (aAD)**

Pitfall traps may be subject of strong disturbance (e.g. by cows) causing some of them be broken. Morover traps are emptied every twenty days from April to October, so that the sum of the collections gives an year sample for each sample site.

To compare data from sites affected by different amount of broken traps, for each species in each site we compute the number of individuals per trap per 10 trapping days as follows:

for every emptying the sampling effort (*sef*) is

$$sef = traps * (dd/10)$$

where *traps* is the number of traps, *dd* the number of actual trapping days

then the total (yearly) sampling effort is

$$SEf = \Sigma\ sef$$

and the annual Activity Density (i.e. the number of individuals per trap per 10 trapping days) is

$$aAD = tot\ indiv\ /\ SEf$$

where *tot indiv* is the total number of individuals captured in that site

If the data of each single emptying should be kept alone, then the Activity Density (*AD*) is computed as follows

$$AD = [indiv\ /\ (traps\ *\ dd)]\ *\ 10$$

where *indiv* is the number of individuals captured in that emptying
